# Diseases of the Blood

**Published:** 1906-05-12

**Authors:** 


### Progress in Medicine and Surgery.

### DISEASES OF THE BLOOD.

**Hypertonus.**—It is more and more evident that greater attention is needed in the diagnosis and treatment of excessive arterial tension. There are many cases besides those of renal disease where the circulation is impeded, the heart overtaxed, and even cerebral hæmorrhage caused by this condition. In one person this may be due to no recognised disease or error of diet, in another to some lung condition, in others to cardiac hypertrophy, and in many, perhaps, to what may be called a fixed habit or setting of the machine originally produced by a disease or hard labour which has long since passed away. W. Russell, indeed, thinks that hypertonus is broadly due either to defective elimination or toxic absorption, but it is hardly fair to assume the existence of toxins of which we know nothing. We may grant that some cases, such as those of kidney disease, are dependent on the failure to eliminate toxins, but in others we have no evidence at all of their presence, while we do know that habit, or unconscious memory, as Creighton called it, may cause permanently a special set of the machine long after the original stimulus has ceased. One effect of hypertension which he notices is the occurrence of sudden paralysis, or paresis, without loss of consciousness, due to spasm of the cerebral vessels. If we can exclude embolism, hæmorrhage, or thrombosis in persons with high tension, these attacks are probably due to such contraction of the cerebral vessels as cuts off the blood supply more or less completely, and these persons will often recover if treated with vasodilators. If the heart is feeble in these cases cardiac stimulants are sometimes necessary as well. Indeed, we are apt to diagnose a feeble heart when it does not really exist, from the small thready pulse produced in contracted vessels. When hyptertension exists together with atheroma of the vessels cerebral hæmorrhage may easily occur, or there may be giddiness and pains in the head. Finally, he would refer many cases of nocturnal restlessness and slight delirium in the aged to the same cause, and finds they are best treated in a similar way or by paraldehyde and a laxative. The effect of tobacco on the blood-pressure has been investigated by Barrazoni amongst others. He concludes that it causes a peripheral vascular contraction, and to some extent strengthens the cardiac systole. The effect of any particular brand of tobacco depends on the proportion of nicotine in it. Women are more affected than men, and habit reduces to some extent the amount of contraction. The action of tobacco on the pulse is almost instantaneous, and a second dose has much less effect. When the smoker is fasting the change in the pulse is greater, and appears and passes off sooner than when the stomach contains food. With low-tension pulses there may be nervous depression and irregular action of the heart, while with high tension there is a very great rise of pressure, and this may cause dyspnœa, vertigo and præcordial distress. Here, too, we are confronted with the question how far the constant use of tobacco leads to permanent high tension. It is clear that a temporary rise is produced, but some authorities are inclined to believe that, quickly as these effects pass off, the frequent repetition of the dose causes a lasting condition of high tension. It is possible that some evidence on the point might be obtained by comparing the blood-pressure of a very large number of non-smokers and of smokers, but there appear to be at present no reliable data on the subject. Among those who look upon hypertonus as due to defective elimination, Haig endeavours to show that the uric acid in the blood slows the circulation in the capillaries and raises the pressure, and that there is a constant relation between the amount of the uric acid present and the pressure. The uric acid, he thinks, acts mechanically, hindering the flow through the capillaries. Parkes Weber, in recent experiments, found that the viscosity of the blood depended in part at least on the number of red cells present, but others have laid down that the lowest viscosity exists when about 5,000,000 per cmm. are present, and that either an excess or defect raises it. In opposition to Haig, a theory has been brought forward by Francis Hare that an excess of unoxydised carbohydrates in the blood leads to vaso-constriction, or at least to a localised constriction. Thus, in migraine he thinks there is a vaso-dilatation of the small affected area with widespread vaso-constriction. The generalised constriction may be thought of as forcing the blood into the small dilated area, so that the distension in the latter is the result of both factors which occur together. In fact, migraine is not unlike angina pectoris, according to certain theories. We have a painful distension of a small part of the arterial system chiefly dependent on a contraction of a large peripheral area. The view certainly explains many phenomena in migraine, such as the different condition of the vessels as to contraction which has been observed, and the effect of various remedies. Thus, relief is often obtained by cold applied to the head, by hot baths for the feet or entire body, by nitrite of amyl, by antipyrin which probably reduces the systolic pressure, or by epistaxis which relieves the local distension. His theory as to the causation of the vaso-contraction by an accumulation of carbonaceous products instead of by the purin bodies must, however, be decided on other grounds.

